# Development and initial feasibility testing of an intergenerational cooking and nutrition program for persons with dementia and young adults: Preliminary results

**DOI:** 10.1002/alz70858_105632

**Published:** 2025-12-26

**Authors:** Kristina Devlin, Sheng Han Liu, Kate Dupuis, Marina Mourtzakis, Sherry L. Dupuis, Heather Keller

**Affiliations:** ^1^ University of Waterloo, Waterloo, ON, Canada; ^2^ Sheridan College, Oakville, ON, Canada; ^3^ Schlegel‐UW Research Institute for Aging, Waterloo, ON, Canada

## Abstract

**Background:**

Intergenerational programs provide an opportunity to connect generations. Currently, there are few community‐based programs that combat dementia stigma, and few food‐based opportunities for connecting community‐dwelling persons living with dementia (PWD) with youth. Cooking Together is a collaboratively designed and tested intergenerational cooking and nutrition program. The aim of this study was to identify and develop components that would be meaningful and acceptable to potential end users (phase 1) and conduct a feasibility test (phase 2).

**Method:**

Phase 1 interviewed (*n* = 11) youth (14‐19 years) and PWD and care partners dyads to inform design of the proposed program and identify potential benefits. The phase 2, 4‐week prototype was tested with 15 participants, who had not participated in phase 1 development. Phase 2 participants included those from a dementia day program (*n* = 8), day program facilitators (*n* = 3), and youth (*n* = 4; 18‐30 years). Data collection included participant observation notes (e.g., engagement, feasibility issues) and interviews (*n* = 10) focused on improvements, participant experience, and potential outcomes. Content analysis was used to summarize findings.

**Result:**

Findings from phase 1 identified important components to include in the prototype for phase 2 testing. For example, potential challenges included daytime scheduling for youth under 18 years. Suggested benefits included socialization for PWD, and dementia education for youth. The phase 2 prototype was found to be feasible. Participants identified potential outcome measures and feedback led to refinement of the model for future feasibility pilot testing.

**Conclusion:**

An intergenerational cooking and nutrition program for community‐dwelling PWD and young adults (18‐30 years) was created and a prototype demonstrated interest in the program and outcomes that should be measured.

